# Publisher Correction to: Protocol of a population-based prospective COVID-19 cohort study Munich, Germany (KoCo19)

**DOI:** 10.1186/s12889-020-09394-x

**Published:** 2020-09-01

**Authors:** Katja Radon, Elmar Saathoff, Michael Pritsch, Jessica Michelle Guggenbühl Noller, Inge Kroidl, Laura Olbrich, Verena Thiel, Max Diefenbach, Friedrich Riess, Felix Forster, Fabian Theis, Andreas Wieser, Michael Hoelscher, the KoCo19 collaboration group, Christiane Fuchs, Abhishek Bakuli, Judith Eckstein, Günter Froeschl, Otto Geisenberger, Christof Geldmacher, Arlett Heiber, Larissa Hoffmann, Kristina Huber, Dafni Metaxa, Michel Pletschette, Camilla Rothe, Mirjam Schunk, Claudia Wallrauch, Thorbjörn Zimmer, Stephan Prückner, Jan Hasenauer, Noemi Castelletti, Eleftheria Zeggini, Michael Laxy, Reiner Leidl, Lars Schwettmann

**Affiliations:** 1 Institute and Outpatient Clinic for Occupational, Social and Environmental Medicine, Ludwig-Maximilians-Universität (LMU) University Hospital Munich, Ziemssenstr. 1, 80336 Munich, Germany; 2 Center for International Health, LMU University Hospital, Munich, Germany; 3 Division of Infectious Diseases and Tropical Medicine, LMU University Hospital Munich, Germany, Leopoldstrasse 5, 80802 Munich, Germany; 4 German Center for Infection Research (DZIF), partner site Munich, Munich, Germany; 5 Helmholtz Zentrum München, Ingolstädter Landstraße 1, 85764 Neuherberg, Germany; 6 Departments of Mathematics and Life Sciences, Technical University of Munich, Munich, Germany; 7 Max von Pettenkofer Institute, Faculty of Medicine, LMU of Munich, Munich, Germany

**Correction to: BMC Public Health 20, 1036 (2020)**

**https://doi.org/10.1186/s12889-020-09164-9**

In the publication of the original article [1], the author Dr. Fuchs was erroneously omitted as a collaborator from the KoCo19 collaboration group.

The collaboration group has been updated above. The original article [1] has been corrected.

The publisher apologizes for the inconvenience caused to the authors and readers.
